# Biological Mechanisms Induced by Soybean Agglutinin Using an Intestinal Cell Model of Monogastric Animals

**DOI:** 10.3389/fvets.2021.639792

**Published:** 2021-06-02

**Authors:** Li Pan, Yan Liu, Hainan Lan, Nan Bao, Yuan Zhao, Hui Sun, Guixin Qin, Mohammed Hamdy Farouk

**Affiliations:** ^1^Key Laboratory of Animal Production, Product Quality and Security, Ministry of Education, Key Laboratory of Animal Nutrition and Feed Science, College of Animal Science and Technology, Jilin Agricultural University, Changchun, China; ^2^Animal Production Department, Faculty of Agriculture, Al-Azhar University, Cairo, Egypt

**Keywords:** soybean agglutinin, cell apoptosis, proteomics, anti-nutritional mechanism, signal pathway

## Abstract

Soybean agglutinin (SBA) has a toxic effect on most animals. The anti-nutritional mechanisms of SBA are not fully understood, in terms of cell survival activity and metabolism of intestinal cells. This study aims to investigate the effects of SBA on the cell cycle, apoptosis, and to verify the mechanism of SBA anti-nutritional characters based on proteomic-based analysis. The IPEC-J2 cell line was cultured with medium containing 0.0, 0.5, or 2.0 mg/mL SBA. With increasing SBA levels, the percentage of the cells at G0/G1 phase, cell apoptosis rates, expressions of Bax and p21, and the activities of Casp-3 and Casp-9 were increased, while cyclin D1 and Bcl-2 expressions were declined (*p* < 0.05). The proteomic analysis showed that the numbers of differentially expressed proteins, induced by SBA, were mainly enriched in different pathways including DNA replication, base excision repair, nucleus excision repair, mismatch repair, amide and peptide biosynthesis, ubiquitin-mediated proteolysis, as well as structures and functions of mitochondria and ribosome. In conclusion, the anti-nutritional mechanism of SBA is a complex cellular process. Such process including DNA related activities; protein synthesis and metabolism; signal-conducting relation; as well as subcellular structure and function. This study provides comprehensive information to understand the toxic mechanism of SBA in monogastrics.

## Introduction

Soybean agglutinin (SBA), also known as lectin, represents about 10% of total protein in mature soybean seeds. Therefore, SBA is a major anti-nutritional factor (ANF) in soya products. SBA is highly resistant to the digestive enzymes and can interact with the mucosal tissue of the digestive tract (1). This ANF has a toxic effect on most monogastric animals, which can lead to reduce the absorption capability, and consequently declines the productive performance of the monogastrics. For example, SBA has a negativeeffect on the digestive system in pigs. The high dose of SBA in pig's diets could increase the output of total nitrogen in ileum, and causes weight loss and diarrhea (2, 3). In order to deeply understand the anti-nutritional principle of SBA, many histopathological, cellular, and molecular researches have been conducted in the last decades. These studies demonstrated that SBA can induce cellular hyperplasia and hypertrophy of the small intestine, thus leading to reduce both feed conversion ratio and growth performance (4). A high dose of SBA could also cause microvillous atrophy in the small intestine (3), with a large number of intestinal epithelial cells shedding into the lumen (5). Studies at the cellular level indicated that SBA could damage the cell-to-cell interaction, increase cell apoptosis rates, inhibit cell proliferation, and affect some signal transduction pathways (6–11).

The molecular investigations have provided more details to understand the principle of SBA toxicity. SBA induces some membranous specific proteins that can change physiological functions of the intestinal epithelial cells. Zhao et al. (2) indicated that a dose of 0.1–0.2% SBA can increase the intestinal permeability and reduce the expressions of occludin and ZO-1 in piglet intestinal epithelium, while 0.05% SBA of total diet had no significant effects. Pan et al. (8) showed that SBA decreases the expression of occludin and claudin-3 in intestinal porcine epithelial cell (IPEC-J2). In addition to tight junction proteins, our previous trials have confirmed that integrins (which has no direct interaction with SBA) were also important to change the cell biological activities, induced by SBA in IPEC-J2. Alpha-actinin-2 (ACTN2) acts as a mediator to connect SBA and integrins. SBA may reduce the mRNA expression of integrins by down-regulating the gene expression level of ACTN2 (11). Although these results can partially explain the mechanism of the anti-nutritional effects of SBA, the full understanding of anti-nutritional mechanism of SBA is still limited, as the SBA-induced cytotoxicity may be involved in a complex process of interactions.

Protein is the executor of physiological function, and a direct embodiment of the life phenomena. Proteomic studies aim to systematically clarify many aspects of functional proteins in the biological system including, most related differentially expressed proteins, and metabolic pathways. Such studies can be helpful for the comprehensive demonstration of the toxic mechanism of SBA on the morphological structure and physiological function of intestinal epithelial cells. Therefore, this research aims to investigate the influences of SBA on cellular biological activities, whole cell proteomic changes, and the correlations between them, using a cell model of monogastric intestinal cells. This could provide more effective information for full understanding the mechanism of SBA toxicity in terms of verifying the mechanism of SBA anti-nutritional characters.

## Materials and Methods

### Experimental Design

The herein investigation was conducted according to the Ethical Guideline of the Jilin Agricultural University.

Recently, IPEC-J2 cell line has been reported as a favorable model to evaluate the intestinal function of the monogastrics (12). Therefore, such promising cell model was selected to be used in the current study. The experiment was randomly divided into three groups (0.0, 0.5, or 2.0 mg/mL SBA), each group with three replicates, and repeated for three times. The treatment time was 24 h (8). The main measured indexes in this experiment included the percentage of the cells at different cell cycle phases, cell apoptosis rates, the protein expressions of cell cycle and apoptosis regulated proteins, the activity of caspase-3 (Casp-3) and caspase-9 (Casp-9), and the whole-cell quantitative proteome analysis.

### Cell Culture

IPEC-J2s were cultured in Dulbecco's Modified Eagle Media: Nutrient Mixture F-12 medium (DMEM/F12) (Gibco, Carlsbad, CA, USA), supplemented with 10% fetal bovine serum (FBS, Gibco, USA) and 1% penicillin-streptomycin (Sigma, USA), incubated at 37°C and in an atmosphere of 5% CO_2_. The medium was refreshed every 2 d and the cells were sub-cultured with 0.05% trypsin (Gibco, USA).

### PI/RNase Staining Analysis

Upon reaching 80% of confluence, the cells were treated with 0.0, 0.5, or 2.0 mg/mL SBA for 24 h. We used propidium iodide (PI) to estimate the percentage of the cells at different cell cycle phases (G0/G1 phase, S phase and G2 phase) and cell apoptosis rates. PI/RNase staining buffer was used for 30 min at 37°C to determine the cell cycle by flow cytometry (FCM). The experiment was carried out according to the manufacturer's instructions (BD Pharmingen, San Diego, CA, USA).

### Determination of Apoptotic Cell Death by Annexin V-FITC/PI Staining

After being treated with 0.0, 0.5, or 2.0 mg/mL SBA for 24 h, the cell apoptosis rates were determined using FITC Annexin V Apoptosis Detection Kit. The procedure was carried out according to the manufacturer's instructions (BD Pharmingen, USA). Data was analyzed using the FlowJo 7.6 software (TreeStar, OR, USA).

### Cell Morphological Observation

IPEC-J2 cells were seeded at 5 × 104 cells/cm^2^ in 6-well-plates and cultured for 24 h at 37°C in an atmosphere of 5% CO_2_ and 95% O_2_. Upon reaching 80% of confluence, the treated cells were morphologically observed by contrast microscopy (×200).

### Cell Protein Extraction

The IPEC-J2 cells were treated with 0.0, 0.5, or 2.0 mg/mL SBA for 24 h, and cell sample was sonicated three times on ice using a high intensity ultrasonic processor (Scientz) in lysis buffer (1% Protease Inhibitor Cocktail, 8 M urea). Then the samples were centrifuged at 12,000 × g at 4°C for 10 min to remove the remaining debris. Finally, we collected the supernatant and determined the protein concentration using BCA kit (Beyotime Institute of Biotechnology, Beijing, China) according to the manufacturer's instructions.

### Western Blotting

After being treated with 0.0, 0.5, or 2.0 mg/mL SBA for 24 h, we collected the extracted total proteins from IPEC-J2 cells. Then, the expressions of Cyclin D1, active p21, Bcl-2, and Bax in different treatments were analyzed using Western blotting (WB).

Proteins were separated in a 12% sodium dodecyl sulfate-polyacrylamide gel electrophoresis (SDS-PAGE) gel and transferred to a polyvinylidene fluoride (PVDF) membrane (Bio-Rad Laboratories, Hercules, CA, USA), which was then incubated in blocking buffer for 2 h. The membrane was subsequently incubated with anti-Cyclin D1 antibodies, anti-active p21 antibodies, anti-Bcl-2 antibodies, and anti-Bax antibodies (PTM Biolabs, Hangzhou, China) overnight at 4°C. Incubation with the horseradish peroxidase (HRP)-conjugated goat-anti-rabbit secondary antibody was then performed for an additional 2 h at room temperature. After washing, the target protein signals on the membrane were visualized by Gel imaging system and analyzed by the Quantity One software version 4.6.2 (Bio-Rad, CA, USA).

### Determination of Caspase Activity

Cells culture supernatants in 0.0, 0.5, or 2.0 mg/mL SBA treatments were collected and centrifugated for 20-min at the speed of 2,000–3,000 rpm. Cell culture supernatants (concentration reached 1 million/mL) were repeated freeze-thaw cycles to damage the cells and to release the intracellular components. Then, the extracts were centrifugated for 20 min at the speed of 2,000–3,000 rpm and the supernatant was removed to detect the activities of caspase 3 (Casp-3) and caspase 9 (Casp-9). The Casp-3 and Casp-9 activities were determined using porcine cysteine protease-3(Casp-3) and protease-9(Casp-9) ELISA kit (FEIYA Biotechnology, Jiangsu, China). The Assay procedure was conducted according to the kit's instructions.

### Trypsin Digestion

In the trypsin digestion experiment, a 5 mM dithiothreitol was used to reduce the protein solution for 30 min at 56°C and a 11 mM iodoacetamide was used to alkylate for 15 min at room temperature in the darkness. Then the protein sample was diluted using 100 mM tetraethylammonium bromide (TEAB) to make the urea concentration <2 M. For the first digestion, trypsin (Promega, Madison, WI, USA) was added at 1:50 trypsin-to-protein mass ratio for overnight and 1:100 trypsin-to-protein mass ratio was used for the second 4 h-digestion.

### TMT/iTRAQ Labeling

After the trypsin digestion experiment, the peptide was desalted by Strata X C18 SPE column (Phenomenex, USA) and vacuum-dried. A 0.5 M TEAB was applied to reconstitute the peptide and the experiment was conducted according to the manufacturer's protocol for TMT kit/iTRAQ kit (Jingjie PTM BioLab, Hangzhou Co., Ltd., China). The experiments were conducted with three replicates for each group.

### HPLC Fractionation

The tryptic peptides were fractionated into fractions by high pH reverse-phase High-performance liquid chromatography (HPLC; EASY-nLC 1200, USA) using Agilent 300 Extend C18 column (5 μm particles, 4.6 mm ID, 250 mm length). Briefly, peptides were firstly separated with a gradient of 8–32% acetonitrile (pH 9.0) over 60 min into 60 fractions. Then, the peptides were combined into 18 fractions and dried by vacuum centrifuging (13).

### LC-MS/MS Analysis

The tryptic peptides were dissolved in 0.1% formic acid (solvent A) and directly loaded onto a home-made reversed-phase analytical column (15-cm length, 75 μm i.d.) for peptide separation. The gradient was consisted of: an increase of the rate from 6 to 23% solvent B (0.1% formic acid in 98% acetonitrile) for 26 min, 23–35% in 8 min, climbing to 80% in 3 min, holding at 80% for 3 min, all at a constant flow rate of 400 nL/min using an EASY-nLC 1000 UPLC system.

The eluted peptides were subjected to NanoSpray Ionization source followed by tandem mass spectrometry (MS/MS) in Q ExactiveTM Plus (Thermo Scientific, Waltham, USA) for analyzing, coupled online to the UPLC. The applied electrospray voltage was 2.0 kV. The scan range was 350–1,800 m/z for full scan, and a resolution for full range mass (intact peptides) scan was set in the Orbitrap at 70,000 resolution. For the MS/MS scans, normalized collision energy (NCE) of 28% was used. The fragments were detected in the Orbitrap at a resolution of 17,500. A data-dependent procedure that alternated between one MS scan followed by 20 MS/MS scans with 15.0s dynamic exclusion. Automatic gain control (AGC) was set at 5E4. Fixed first mass was set as 100 m/z.

### Database Search

To identify protein and succinylation, data were processed by the MaxQuant that match Tandem mass search engine (v.1.5.2.8) (14, 15). Tandem mass spectra were searched against the transcriptome data, which was downloaded from the published database (UniProt) concatenated with reverse decoy database.

Carbamidomethyl on Cys was specified as fixed modification and oxidation on Met, acetylation on Lys, and acetylation on protein N-terminal were specified as variable modifications. False discovery rate (FDR) thresholds for protein, peptide and modification site were adjusted to <1%, while minimum score for peptides was set at >40.

### Bioinformatics Method

#### GO Annotation

Gene Ontology (GO) annotation proteome was originated from the UniProt-GOA database (www. http://www.ebi.ac.uk/GOA/). The identified protein ID was firstly converted to the UniProt ID and then was mapped to GO IDs by protein ID. The InterProScan software (versionv.5.14–53.0, http://www.ebi.ac.uk/interpro/) was used to annotated protein's GO functional based on protein sequence alignment method, if some identified proteins were not found in UniProt-GOA database. Finally, the proteins were classified by GO annotation, based on biological process, molecular function, and cellular component (16).

#### KEGG Pathway Annotation

Kyoto Encyclopedia of Genes and Genomes (KEGG) database was used to annotate protein pathway (17). Firstly, protein's KEGG database description was annotated by KEGG online service tools KAAS (KAAS v.2.0, https://www.genome.jp/kaas-bin/kaas_main). Then, KEGG online service tools KEGG mapper was used for mapping the annotation result of the KEGG pathway database.

### Statistical Analysis

Each experiment was repeated at least for three times and numerical data are presented as mean ± standard error of the mean (SEM). Student's *t*-test was used to compare the data between two groups. Data among three groups were analyzed using ANOVA followed by the least significant difference (LSD) tests of SPSS Statistics Base 17.0. Principal component analysis (PCA) was carried out with the RStudio in R v.3.5.3 (18). *p* < 0.05 was considered significant.

## Results

### The Changes of Cell Cycle and Apoptosis in Different SBA Treatments

The effects of different concentrations (0.0, 0.5, and 2.0 mg/mL) of SBA on IPEC-J2 cell cycle progression and cell apoptosis rates were analyzed using flow cytometry (FCM). The percentage of the cells at different cell cycle phases was determined using PI/RNase Staining and the apoptosis rates in different SBA treatments (DST) was evaluated by Annexin V-FITC/PI staining.

**Figure 1:**
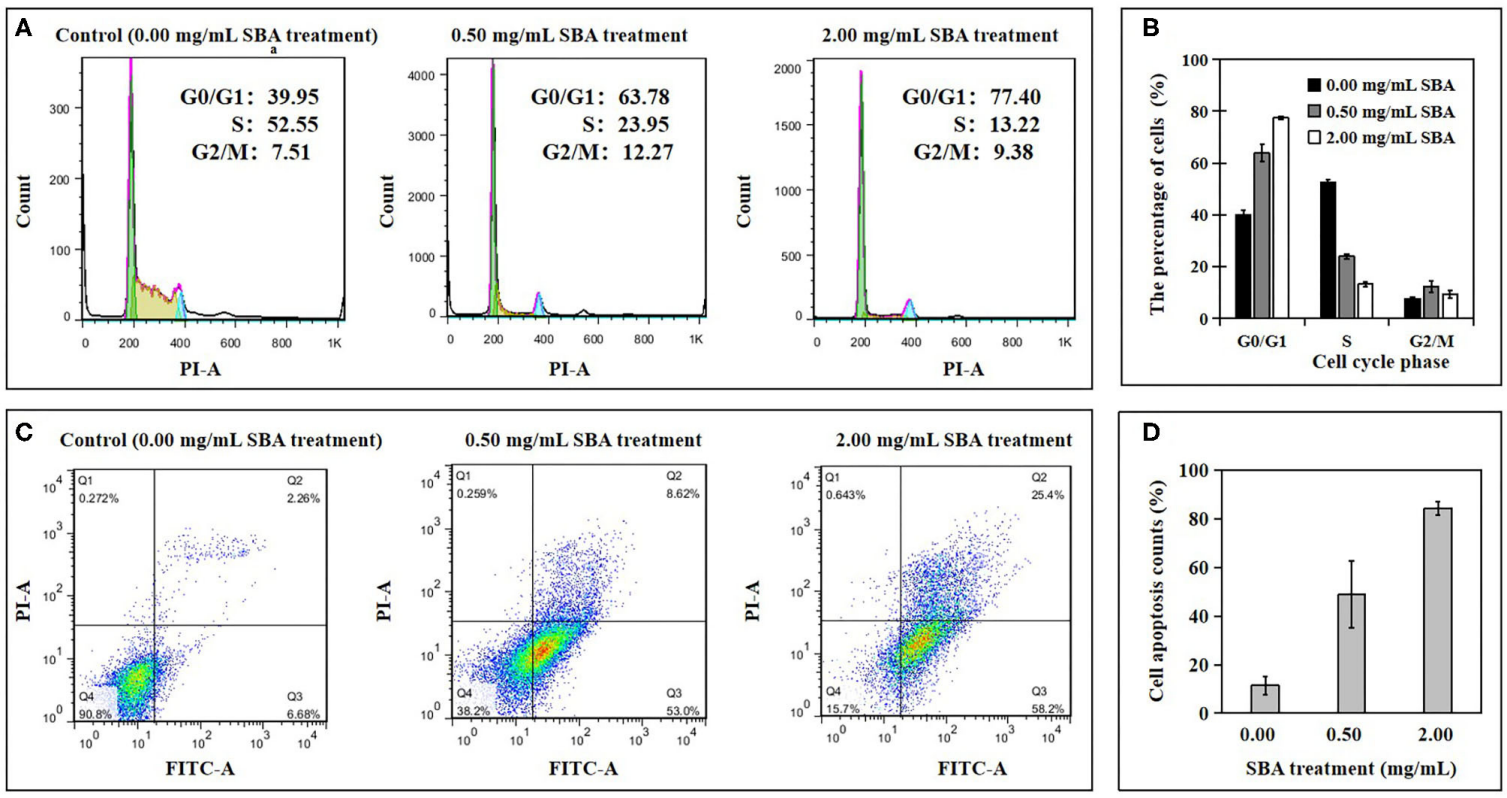
The percentage of the cells at different cell cycle phases and apoptosis rates in DST. After being treated with 0.0, 0.5, or 2.0 mg/mL SBA for 24 h, the cell cycle and cell apoptosis in DST were evaluated using FCM. Cell cycle **(A)** and its analysis **(B)**, cell apoptosis **(C)**, and its analysis **(D)**.

The cell cycle results from FCM showed a significant (*p* < 0.05) delay in the G0/G1 to S phase transition in 0.5 and 2.0 mg/mL SBA treatments, compared to the control (0.0 mg/mL SBA treatment (Figures 1A,B). This result indicated that cell's G0/G1 phase was arrested by SBA. Moreover, the effects of 2.0 mg/mL SBA treatment on the cell cycle progression was more significant (*p* < 0.05) than 0.5 mg/mL SBA treatment.

The apoptotic results indicated a significant increase in apoptotic rates of IPEC-J2 cells for 0.5 and 2.0 mg/mL SBA treatments, compared to control (*p* < 0.05, Figures 1C,D). The cell apoptosis rates in 2.0 mg/mL SBA treatment were higher than 0.5 mg/mL SBA treatment (*p* < 0.05).

**Figure 2:**
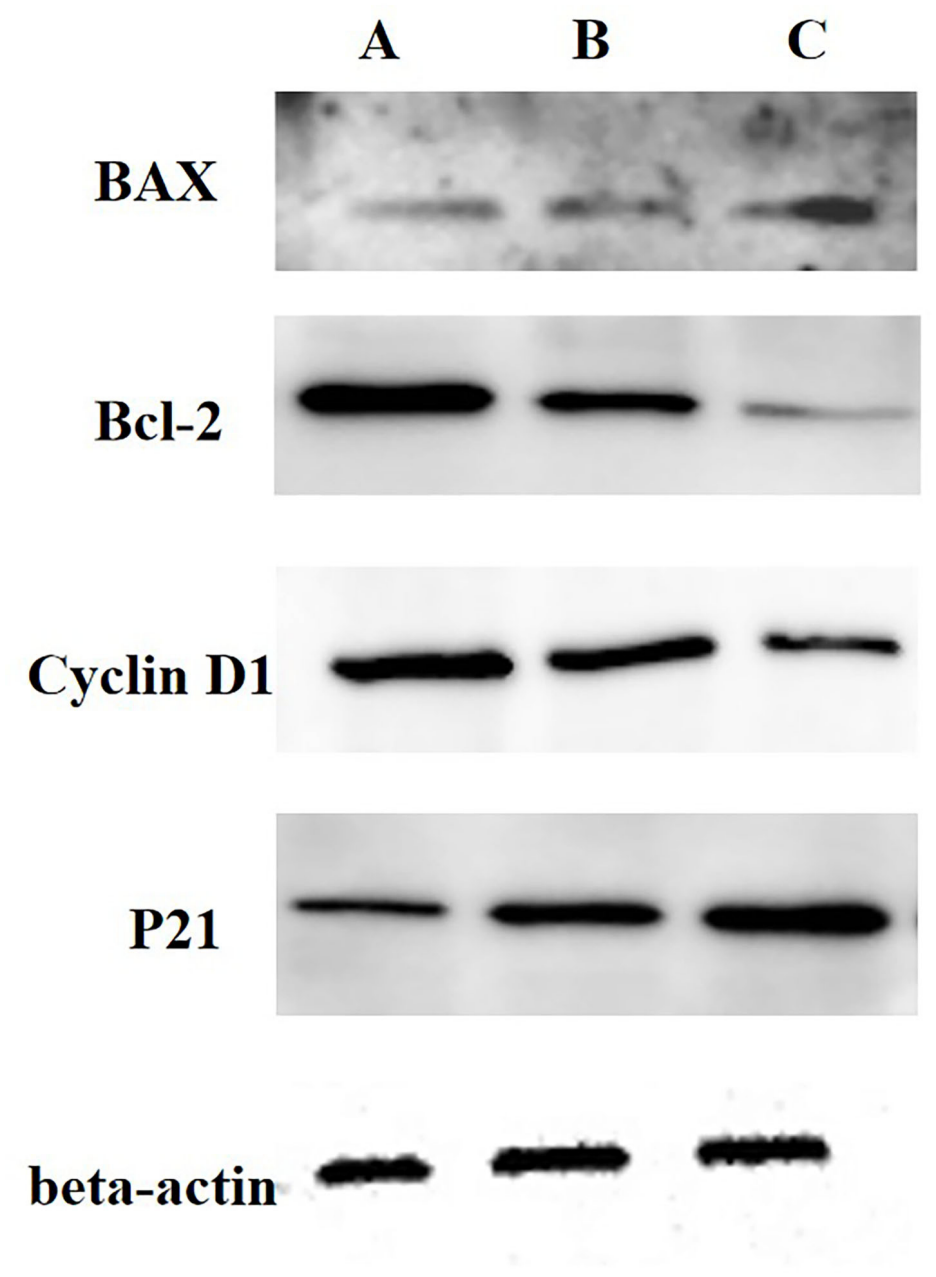
Analysis of Cyclin D1, active p21, Bcl-2, and Bax expressions in DST using WB. IPEC-J2s were treated with 0.0, 0.5, or 2.0 mg/mL SBA for 24 h. **(A)** control, 0.0 mg/mL SBA treatment; **(B)** 0.5 mg/mL SBA treatment; **(C)** 2.0 mg/mL SBA treatment.

**Figure 3:**
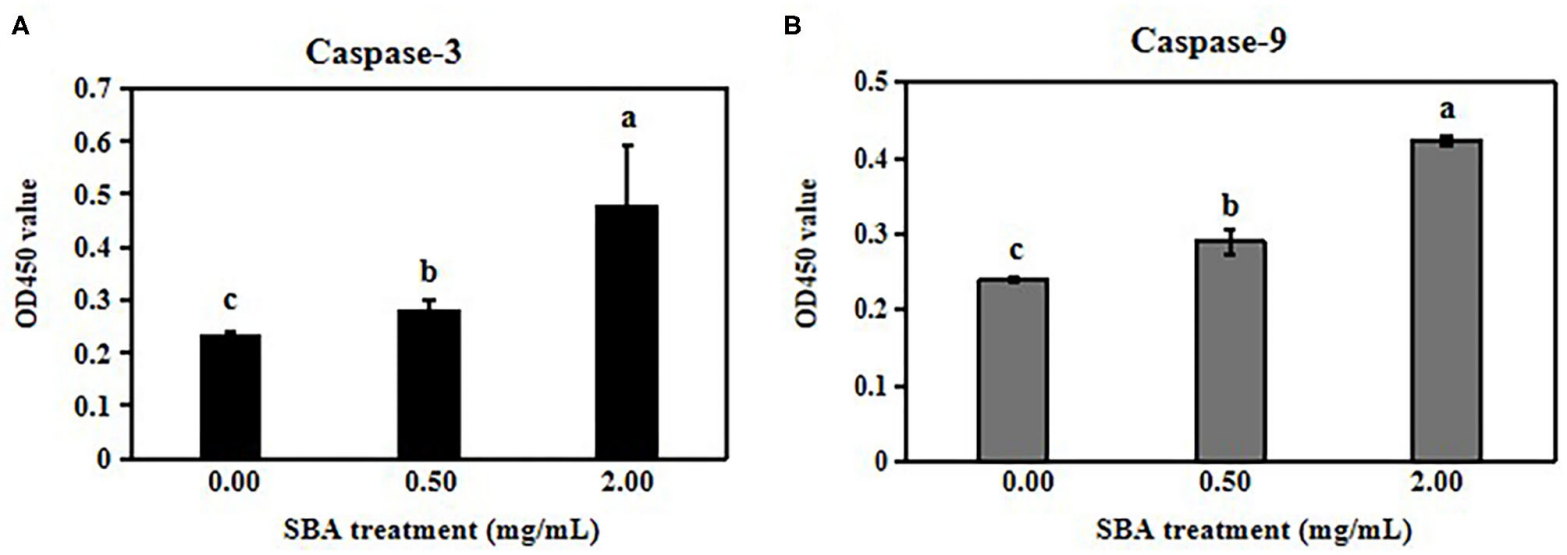
Activation of Casp-3 and Casp-9 by SBA. IPEC-J2 cells were treated with 0.0, 0.5, or 2.0 mg/mL SBA for 24 h. The cells in different treatments were collected and the activities of the Casp-3 **(A)** and Casp-9 **(B)** were determined using ELISA. Data are represented as means ± standard error of the mean (SEM) from three independent experiments, relative to control. Different lowercase letters (a, b, c) represent significant differences among groups (*p* < 0.05).

Then, we analyzed the expressions of cyclin D1, active p21, Bcl-2 and Bax using WB, and we determined the activities of Casp-3 and Casp-9 with ELISA kits. The results showed decreases in the cyclin D1 and Bcl-2 protein expression levels and increases in the active p21 and Bax expressions in 0.5 and 2.0 mg/mL SBA treatments. Additionally, the effect degree of 2.0 mg/mL SBA on the cell apoptosis rates was more significant than 0.5 mg/mL SBA treatment (*p* < 0.05) as shown in Figure 2. Casp-3 and Casp-9 activities are markers for the cells undergoing apoptosis. The results of caspase enzymes indicated significant increases in Casp-3 and Casp-9-like activities in both 0.5 and 2.0 mg/mL SBA treatments (*p* < 0.05). With increasing SBA concentration, the caspase activities were gradually increased (*p* < 0.05, Figure 3). These results also indicated that SBA can lead to mitochondrial outer membrane damage.

### The Cell Morphology in DST of IPEC-J2

**Figure 4:**
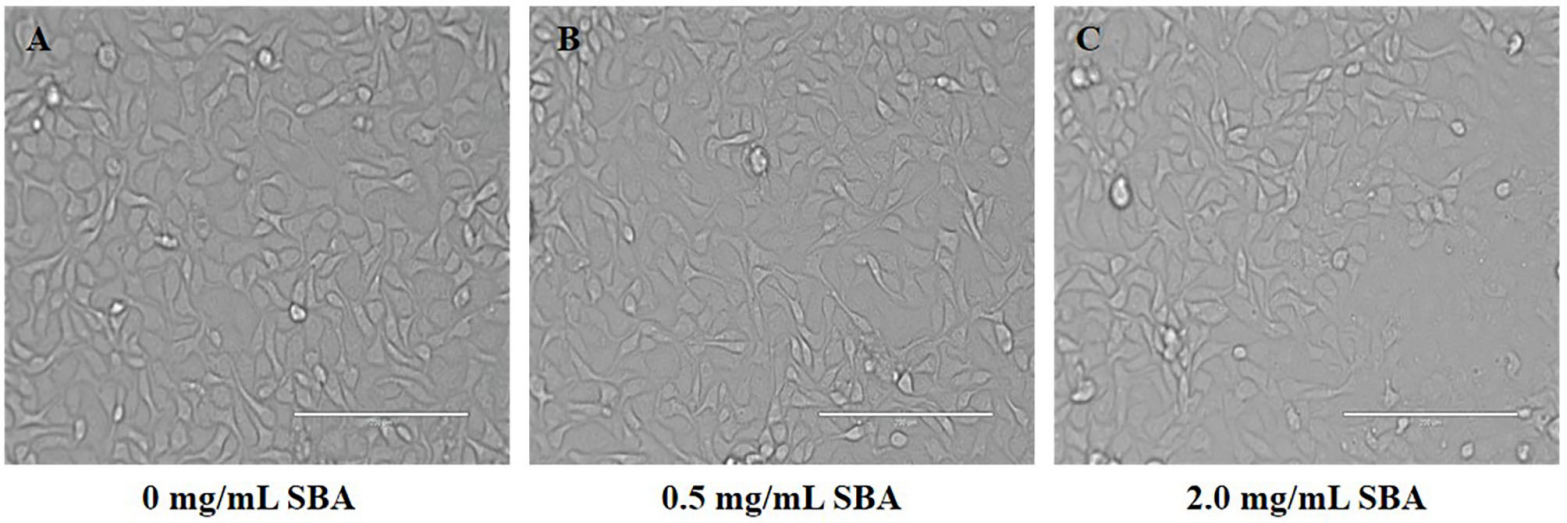
Effect of different SBA concentrations on IPEC-J2 cell morphology (200×). IPEC-J2 was cultured with 0.0, 0.5, or 2.0 mg/mL SBA for 24 h. Cell morphology was observed in different treatments by contrast microscopy at 200× magnifications. **(A)** Control, 0.0 mg/mL SBA treatment; **(B)** 0.5 mg/mL SBA treatment; and **(C)** 2.0 mg/mL SBA treatment.

The results of cell morphology in DST showed that the boundaries between adjacent cells were ambiguous and the cell structure was destructed. Moreover, with increasing SBA concentration, the destruction efficiency of the cells was significantly increased (Figure 4).

### The Whole-Cell Proteomic Comparison Among DST in IPEC-J2

Many cellular biological functions of IPEC-J2 such as cell cycle, cell apoptosis, and morphology were altered in DST and there were fundamental effects for the SBA levels on the cell biological activities. In order to further analyze the mechanisms for the above results, we carried out the whole-cell proteomic test to explain in detail the functions and characteristics of the differentially expressed proteins.

**Figure 5:**
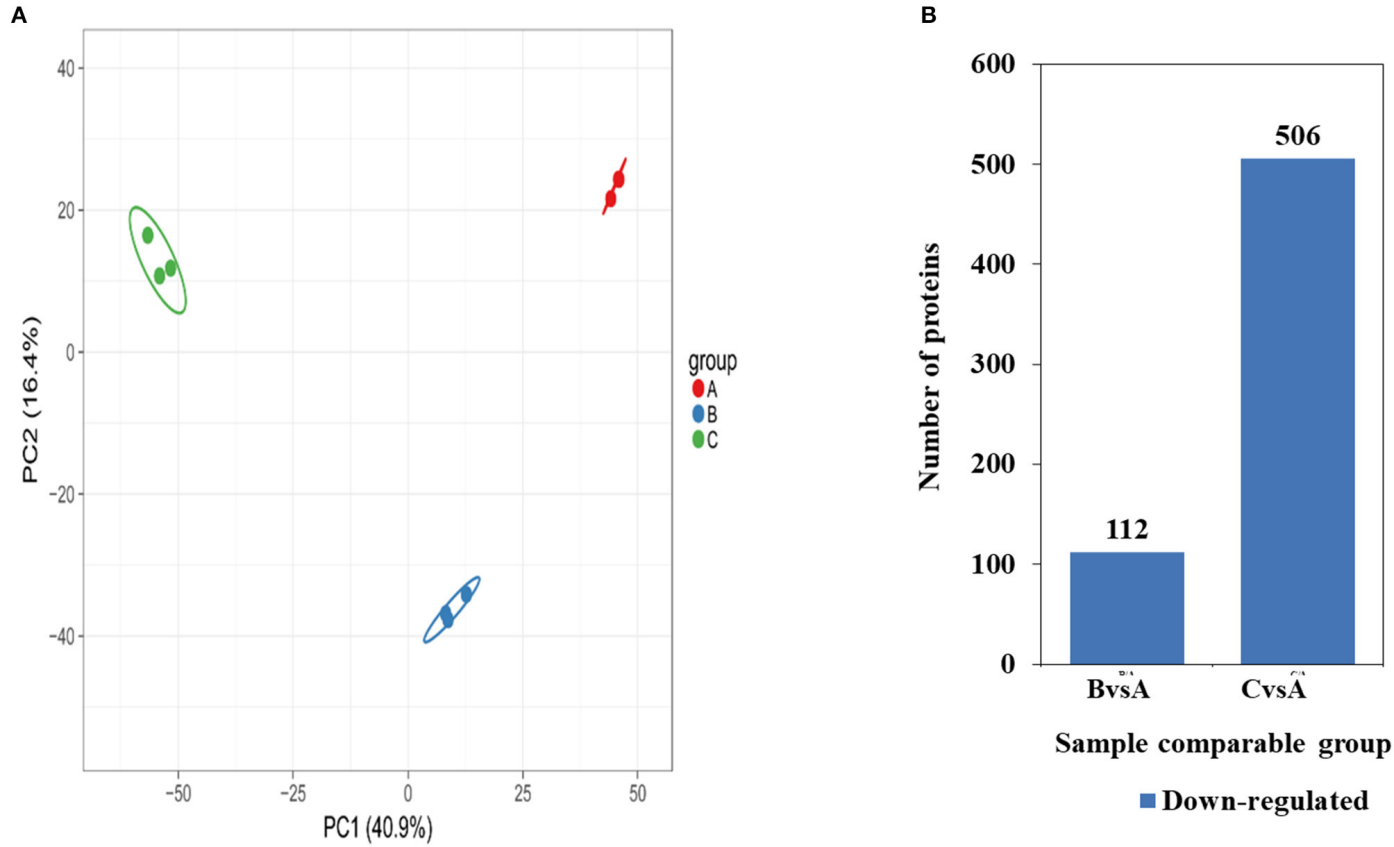
Differentially expressed proteins identified among SBA treatments. **(A)** Two-dimensional scatter plot of PCA (principal component analysis) distribution of all samples using quantified protein in different treatments, **(B)** Up- or down-regulated proteins in different SBA treated groups. Up-regulated proteins are shown in red, down-regulated proteins are shown in blue. BvsA indicates the differential proteins enriched in 0.5 mg/mL SBA treatment when compared to control (0.0 mg/mL SBA treatment), CvsA indicates the differential proteins enriched in 2.0 mg/mL SBA treatment when compared to control.

Based on the proteomics approach, a total of 4,681 quantifiable proteins were identified. To evaluate the significant differences of these proteins, the criteria of *p* < 0.05 and a fold change >1.3 fold were considered significantly differentially expressed. The results of protein quantitative principal component analysis of all samples are shown in Figure 5A. In general, a total of 60 up-regulated proteins and 112 down-regulated proteins were detected in 0.5 mg/mL SBA treatment. Also, 183 up-regulated proteins and 506 down-regulated proteins were detected in 2.0 mg/mL SBA treatment, compared to control (0.0 mg/mL SBA treatment, *p* < 0.05), as shown in Figure 5B.

#### GO Enrichment

**Figure 6:**
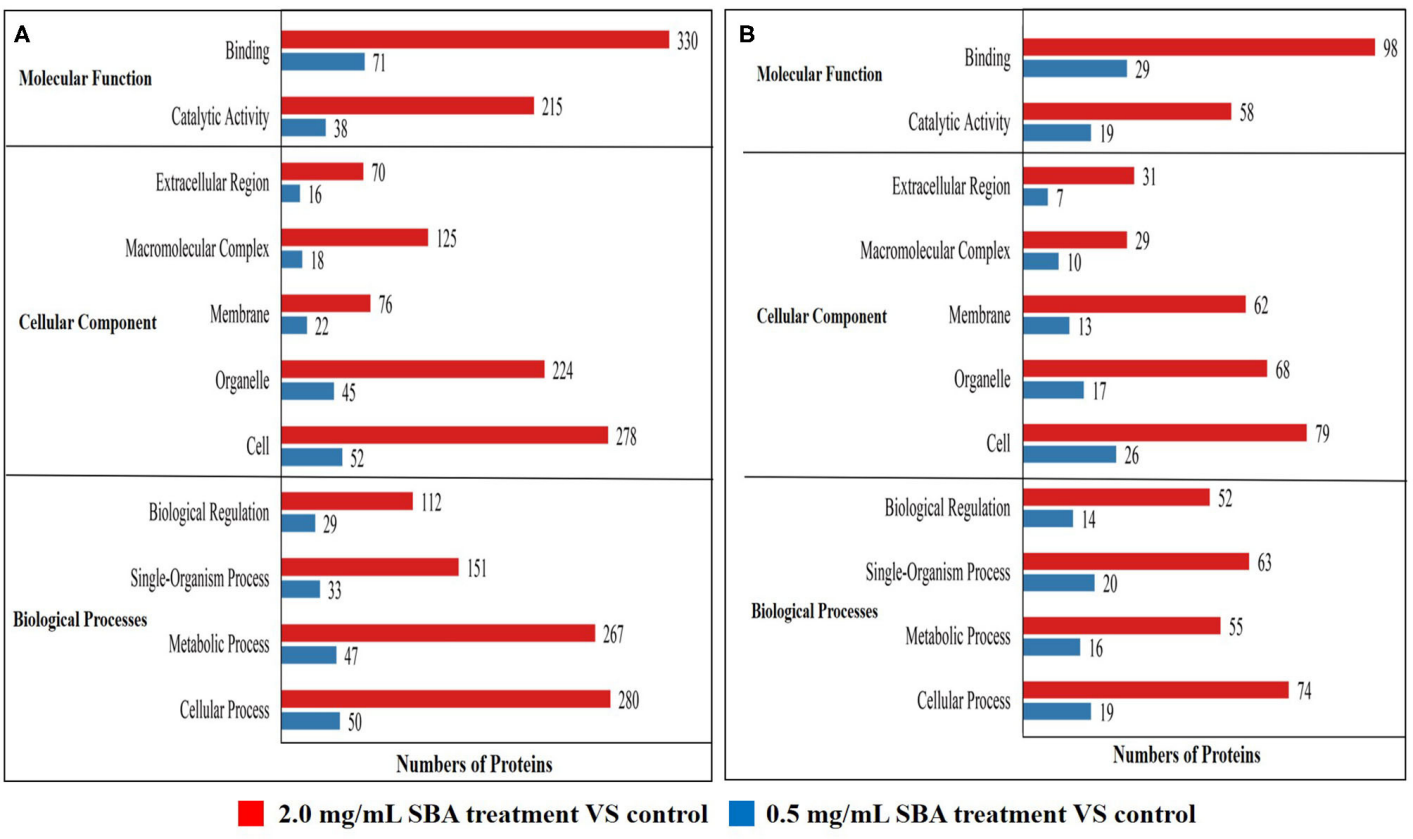
Statistical distribution chart of differentially expressed proteins under each GO enrichment. Enrichment analysis was performed based on biological process, cellular component and molecular function. **(A)** The enriched down-regulated proteins analysis based on biological process, cellular component, and molecular function; **(B)** The enriched up-regulated proteins analysis based on biological process, cellular component, and molecular function.

Among these differentially expressed proteins, we mainly focused on the analysis of the differential proteins related to cell biological activities, including cell cycle, apoptosis, and morphology. The results of GO enrichment analysis showed that the differentially expressed proteins (down-regulated proteins and up-regulated proteins) in 0.5 and 2.0 mg/mL SBA treatments were significantly enriched in biological processes, cellular component, and molecular function, compared to control (0.0 mg/mL SBA treatment, Figure 6). For the molecular function, these proteins were mainly associated with binding and catalytic activity. For the cellular component, these proteins were mainly enriched in extracellular region, macromolecular complex, membrane, organelle, and cell. For the biological process, the proteins were mainly enriched in biological regulation, single-organism process, metabolic process, and cellular process. Specially, the proteins enriched in these three processes were more abundant in 2.0 mg/mL SBA treatment, indicating the damage degree of high-dose SBA to cell biological activities.

##### Cluster Analysis of the Enrichment Patterns of GO Functional Categories Related to Cell Biological Activities

**Figure 7:**
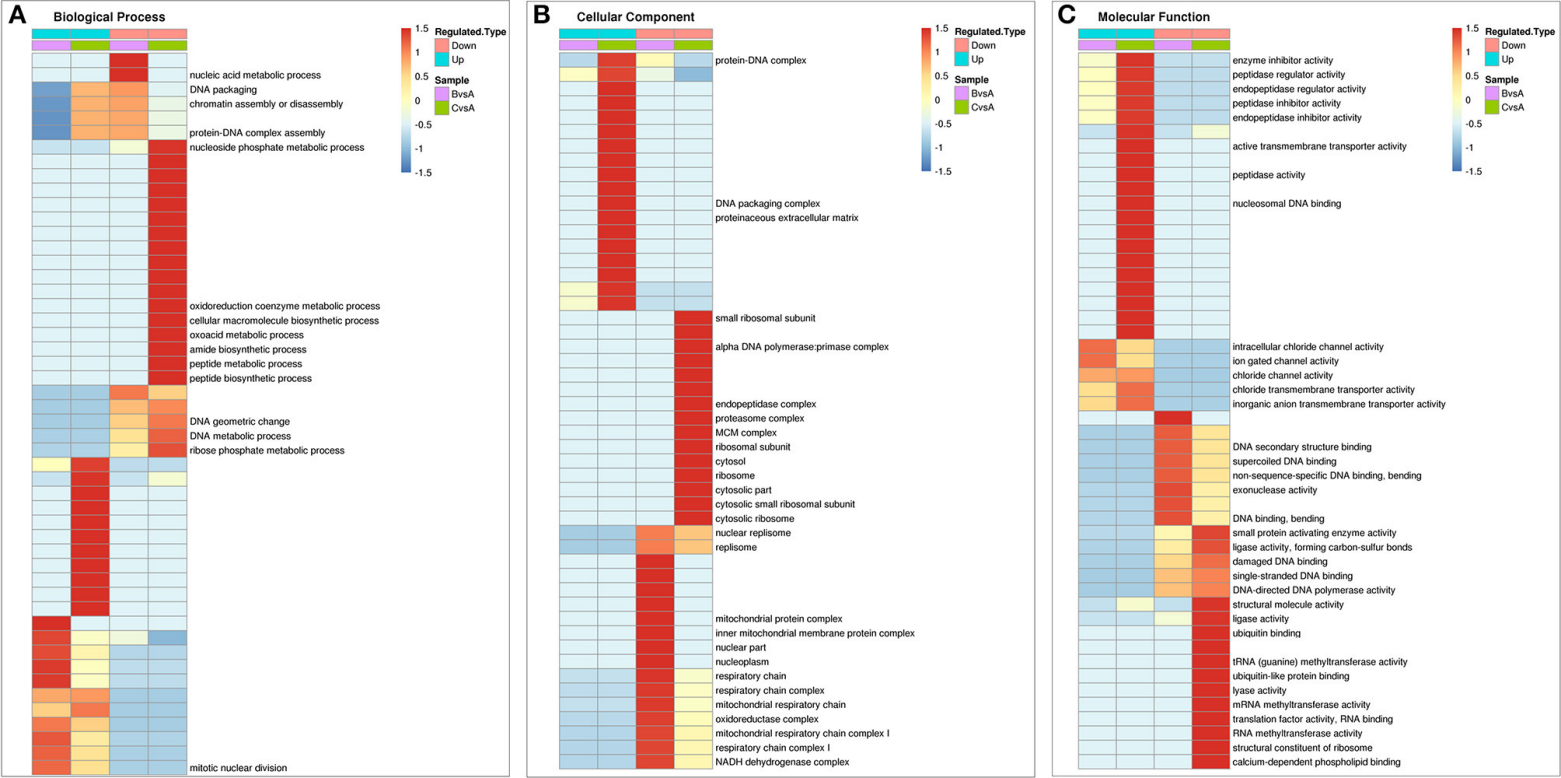
Comprehensive heatmap for cluster analysis of the enrichment patterns of GO functional categories related to cell apoptosis in IPEC-J2 cells. **(A)** Biological process; **(B)** Cellular component; **(C)** Molecular function. (BvsA) indicates the differential proteins enriched in 0.5 mg/mL SBA treatment when compared to control (0.0 mg/mL SBA treatment), (CvsA) indicates the differential proteins enriched in 2.0 mg/mL SBA treatment when compared to control.

For further study the expression patterns of the differentially expressed proteins, clustering analysis of these proteins that related to cell survival activities were performed based on the GO functional categories as shown in Figures 7A–C.

For the details of the biological process, the differential down regulated proteins in 0.5 mg/mL SBA treatment were enriched in nucleic acid metabolism [such as NADH dehydrogenase (F1S031, NDUFS6), Guanine monophosphate synthase (GMPS, A0A286ZY37)], DNA packing, and chromatin assembly [High mobility group protein (HMGB1, F2Z594); Sialic acid synthase (NANS, F1SSG6)]. In 2.0 mg/mL SBA treatment, the differentially expressed proteins were enriched in DNA metabolism [DNA (apurinic or apyrimidinic site) lyase (APEX1, A0A287BTC2)]; Replication protein A (RPA, F1STM9); High mobility group protein B1 (HMGB1, F2Z594), amide biosynthesis such as 40S ribosomal protein S12 (RPS12, P46405); Ribosomal protein S27a (RPS27A, A0A287AZA7); 40S ribosomal protein S28 (RPS28, Q6QAT1); Tryptophan–tRNA ligase (WARS, K9IVV5), peptide biosynthesis [such as RPS27; Elongation factor 1-beta (EEF1B2, F1SHD6); Seryl-tRNA synthetase (SARS, F1S5Z3); RPS28], and other metabolic processes (Figure 7A).

In cellular component classification, the differential down-regulated proteins were mainly enriched in oxidative metabolism of mitochondria such as mitochondrial protein complex and mitochondrial respiratory chain complex [such as succinate dehydrogenase ubiquinone iron-sulfur subunit (SDHB, I3LDC1); NADH dehydrogenase ubiquinone iron-sulfur protein 6 (NDUFS6, F1S031)] in 0.5 mg/mL SBA treatment. While the down-regulated expressed proteins were mainly enriched in the ribosome structural and functional proteins [such as RPS12; 60S ribosomal protein (RPL4, A0A287AE76); N(alpha)-acetyltransferase 10 (NAA 10, F1RZU5); cytoplasmic FMR1-interacting protein 2 (CYFIP) and FMR1 interacting protein 2 (FIP2, F1RN89)], only found in 2.0 mg/mL SBA treatment (Figure 7B).

In the enrichment analysis of differential proteins based on molecular function, the same down-regulated proteins were enriched in both 0.5 and 2.0 mg/mL SBA treatments. Such proteins were presented in DNA synthesis and related kinases activities including, ligase [such as DNA ligase (ATP) activity (PARP3, I3LJ55); DNA ligase (LIG1, F1RL99), cytokine activities (high mobility group protein B1 (HMGB1 F2Z594); and cytokine receptor like factor 1 (CRLF1, F1S909)]. The differences between these two treatments were found in many down-regulated proteins, including endopeptidase activity [proteasome subunit alpha (PSMA1, F2Z5L7); proteasome subunit beta type-7 (PSMB7, A1XQU1); and proteasome subunit beta type-10 (PSMB10, A0A287A509)], tRNA methyltransferase activity [tRNA methyltransferase 2 homolog A (TRMT2A, F1RHM9); tRNA (guanine-N(7)-)-methyltransferase non-catalytic subunit (WDR4, I3LBJ3); and tRNA (guanine(37)-N1)-methyltransferase (TRMT5, I3L8T3)], structural constituent of ribosome and ubiquitin binding proteins (such as RPS12; RPL4; NAA 10; and FIP2) that were only enriched in 2.0 mg/mL SBA treatment. Additionally, the up-regulated proteins related to channel protein activity were enriched in both SBA treatments. Some other proteins were only enriched in 2.0 mg/mL SBA treatment, including enzyme inhibitor activity [such as metalloproteinase inhibitor 2 precursor (TIMP-2, C0JPM4); and serine protease inhibitor 9 (PI-9, A0PA01)], peptidase regulator and inhibitor activities [serpin H1 precursor (SERPINH1, A0A286ZRU9); serpin family F member 2 (SERPINF2, A0A287AJI4)], endopeptidase regulator and inhibitor activities (such as PI-9; TIMP-2; SERPINH1) (Figure 7C).

### KEGG Enrichment Analysis

**Figure 8:**
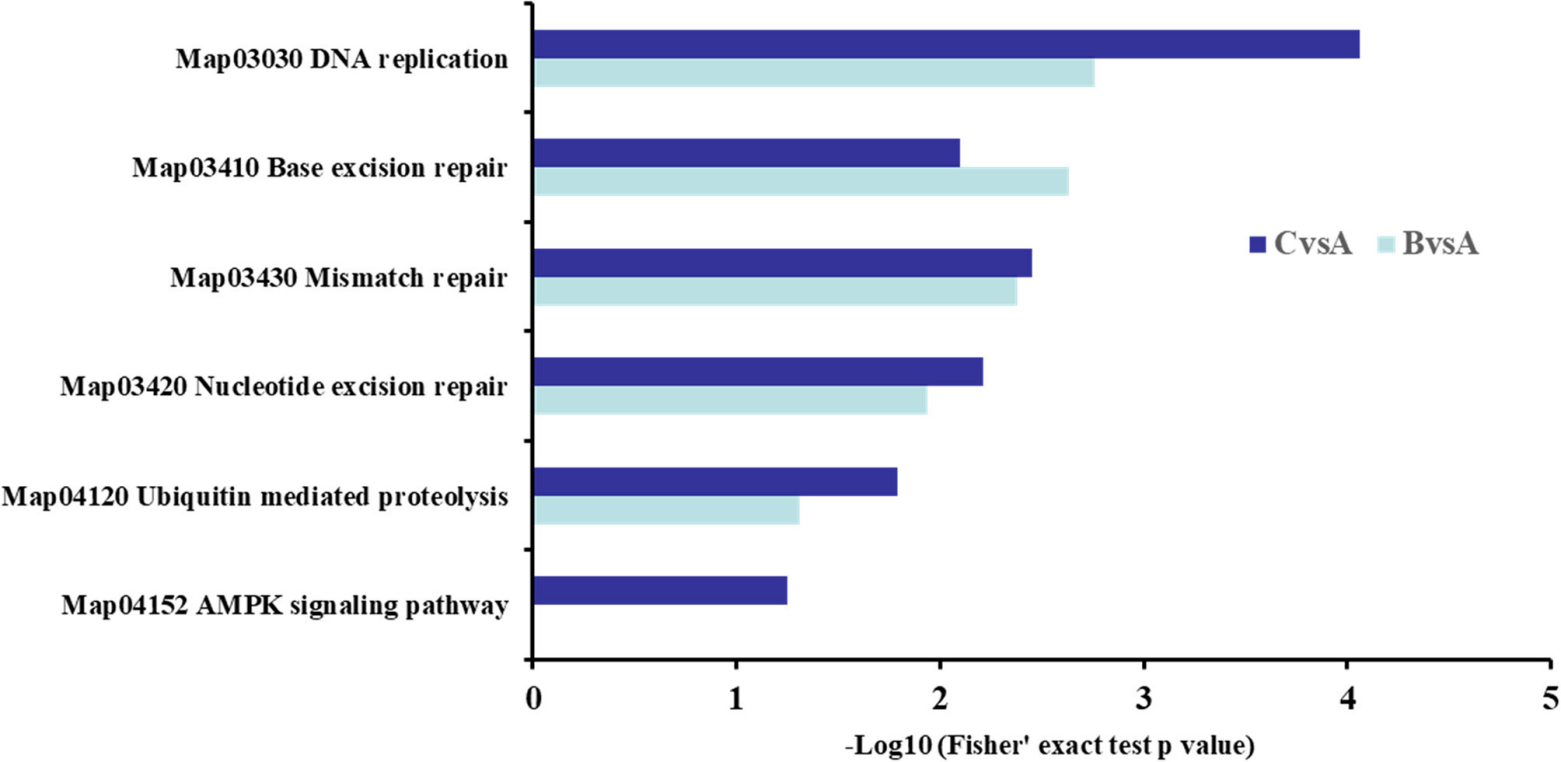
KEGG analysis in DST. BvsA indicated the differential proteins enriched in 0.5 mg/mL SBA treatment compared to control (0.0 mg/mL SBA treatment), CvsA indicated the differential proteins enriched in 2.0 mg/mL SBA treatment compared to control.

The KEGG enrichment analysis of DST showed that the apoptotic-related proteins were more abundant in 2.0 mg/mL SBA treatment. In addition to the cell cycle and apoptosis pathway mentioned above, the differentially expressed proteins were mainly enriched in DNA replication, base excision repair, nucleotide excision repair, mismatch repair, and ubiquitin mediated proteolysis in both SBA treatments. In addition, the AMP-activated protein kinase (AMPK) signaling pathway was only enriched in 2.0 mg/mL SBA treatment compared to the control group (Figure 8).

The key regulated proteins in DNA replication, base excision repair, nucleotide excision repair, and mismatch repair pathway, including proliferating cell nuclear antigen (PCNA, A0A287ABG2), DNA polymerase δ1 (POLD1, A0A287B7N1), DNA polymerase α1 (POLA1, A0A287APS7), and replication protein A (RPA, F1STM9) that were significantly reduced in both SBA treatments. The reduction was more significant in the 2.0 mg/mL SBA treatment. Moreover, with increasing the concentration of SBA, the protein expressions of cell proliferation-related pathway were decreased significantly. In addition, the protein types were increased in the same signal pathway proteins in 2.0 mg/mL SBA treatment. Therefore, some other proteins that were specially enriched in 2.0 mg/mL SBA treatment included RNA processing and modification, replication, recombination and repair like DNA ligase (Lig, F1RL99), DNA helicase (MCM 2, F1SPF3; MCM 4, F1RSE7; MCM 5, I3LR86), and DNA mismatch repair proteins (MSH2, F1SQH4).

In the ubiquitin mediated proteolysis, the down-regulated ubiquitin activating enzymes E1 (UBLE1A, F1RM03; UBLE1B, A0A287AML4) were enriched in 0.5 mg/mL SBA treatment, while other proteins were enriched in 2.0 mg/mL SBA treatment, including ubiquitin activating enzyme E1 (UBE1, K7GRY0; UBLE1A, F1RM03; UBLE1B, A0A287AML4; UBE1C, A0A287A4L4), and ubiquitin-conjugating enzyme E2 (UBE2I, I3LSZ1; UBE2L3, B8Y648; UBE2M, A0A287BNE4; UBE2N, F1SQ14), compared to control. In the high concentration of SBA (2.0 mg/mL) treatment, there were many proteins that mainly enriched in AMPK signal pathway, including down-regulated proteins, sirtuin 1 (SIRT1, A7LKB1), serine/threonine-protein phosphatase 2A (PP2A, I3LGC0), fatty acid synthase (FASN, I3LC73), acyl-coA desaturase (SCD, Q6RWA7), acetyl-ccoA carboxylase (ACC, D2D0D8), eukaryotic translation elongation factor 2 (eEF2, A0A287A1E0), and up-regulated protein carnitine o-palmitoyltransferase (CPT1, F1RY67).

## Discussion

In this research, cell cycle G0/G1 phase arrest and the increase of cell apoptosis rates were observed with increasing levels of SBA. The proteomic analysis showed that SBA led to identify numbers of differentially expressed proteins that were mainly enriched in DNA-related biological processes, protein translation and metabolism, signal-conducting relation, as well as subcellular structure and function.

### SBA Induced Cell Cycle G0/G1 Phase Arrest and Increased Cell Apoptosis Rates

The cell cycle progression regulates the condition of cell proliferation, which consists of three prominent phases to maintain DNA integrity (19). In the herein study, the percentage of cells at G0/G1 phase were increased in SBA treatments, and the G1-phase cells that reflect the high number of cells in the DNA repairing process. Bakke-McKellep et al. (20) also found the same trends in Atlantic Salmon. In addition to FCM analysis, WB was performed to detect the expression of cell cycle key regulatory proteins (p21 and cyclin D1). The p21 protein belongs to the Cip/Kip family of CDK (cyclin-dependent kinase) inhibitors. Such protein is an important cell cycle regulator (21). Loss of functional p21 along with p53 may lead to promote cell cycle progression into the S phase, despite the presence of DNA damage (22). Cyclin D1 is involved in the G1/S cell cycle progression, and the role of cyclin D1 seems to be opposite to p21 (23). Cyclin D1 plays two opposing roles in the cell proliferation (21), the cell cycle arrest, or both biological processes (24).

In the herein research, cell apoptotic rate was determined using FCM, and the cell apoptotic regulatory proteins (Bcl-2 and Bax) were estimated using WB. The results showed that SBA increased cell apoptotic rates, down-regulated the Bcl-2, up-regulated the Bax, and increased the level of cleaved caspases (including casp-9 and casp-3 activities). Yang et al. (25) reported that the abnormal expression of the anti-apoptotic members (Bcl-2) and pro-apoptotic members (Bax) induces the apoptosis. The apoptosome (protein formed during the apoptosis) activates casp-9, which triggers the activation of casp-3 (26, 27). The latter activation initiates proteolytic action which leads to cell death (26, 27).

### SBA Interfered With DNA-Related Biological Processes

The proteomic results of the current study indicated that, after being treated with SBA, the expressions of many proteins were significantly reduced, including PCNA, POLD1, POLA1, and RPA. These reduced proteins play crucial roles in different pathways such as DNA replication, base excision repair, nucleotide excision repair, and mismatch repair. Moreover, the expressions of these four proteins were markedly decreased in the 2.0 mg/mL SBA treatment. Related researches showed that the expression of PCNA (known as cyclin) is necessary for cell proliferation and DNA synthesis (28), which reached its maximum level during the S-phase. The PCNA acts as an auxiliary protein for POLD1 in DNA synthesis (29). Since POLD1 represents one of the three B DNA polymerase families in eukaryotes. This family possesses a crucial role in leading- and lagging-strand synthesis (30–32). Furthermore, POLD1 acts in several aspects of DNA synthesis and DNA-repair processes (33, 34). The POLD1 complex coordinately interacts with a number of proteins that enable its function, such as DNA replication factor C (RFC) and PCNA (35). While, POLA1 protein family plays essential roles in pyrimidine or purine metabolism. POLA1 together with PCNA, are key players in DNA replication during S phase of the cell cycle. The down-regulation of these proteins suppresses the cell cycle, especially DNA replication (36). RPA acts as a laxative to keep the DNA in a regular form and dynamically regulates mono-ubiquitination of PCNA (37, 38). In addition to the above four proteins, Lig, MCM 2, MCM 4, MCM 5, and MSH2 were significantly enriched in 2.0 mg/mL SBA treatment.

In addition, there are relationships among these detected cell-cycle regulatory proteins. For example, Ando et al. (39) and Cayrol et al. (40) and suggested that p21 interacts with PCNA and blocks cell cycle progression. Cazzalini et al. (41) indicated that p21 can prevent or replace the binding of polymerase δ to PCNA at the G1/S phase transition. Sheng et al. (42) showed that PCNA-mediated degradation of p21 can coordinate the DNA damage response and cell cycle regulation.

### SBA Caused Abnormal Protein Synthesis

The proteomics results indicated that the differential expressed proteins were also enriched in amide biosynthesis (such as RPS28; RPS12; RPS27A; and WARS), and peptide biosynthetic and metabolic process (such as RPS28; RPS27A; EEF1B2; SARS; PSMA1; PSMB7; PSMB10; TRMT2A; TRMT5 etc) in 2.0 mg/mL SBA treatment. RPS28 is a component of the 40S ribosome, which is essential for the biogenesis of 18S rRNA (43). The importance of the RPS28 protein in translation can be inferred from its localization to the head of the small ribosomal subunit. The limitation of RPS28 can cause a detrimental effect on translation, making cell death a preferred outcome (44). RPS27A protein performs extra-ribosomal functions in addition to its role in ribosome biogenesis and post-translational modifications of proteins. RPS27A can promote cell proliferation, regulate cell cycle progression, and inhibit the apoptosis of leukemia cells (45). PSMB7 is critical for proteasome assembly (46), and to mitigate endoplasmic reticulum stresses (47). TRMT2A has a domain related to RNA methyltransferase (48). In addition, TRMT2A was a novel open reading frame whose expression varies during the cell cycle and classic proliferation markers (49).

The differential expressed proteins were also enriched in the ubiquitin-activating enzyme E1 and ubiquitin-conjugating enzyme E2. Such enzymes are members of the ubiquitin mediated proteolysis system. Ubiquitin-mediated proteolysis system acts in broad array of basic cellular processes, including regulation of the cell cycle, differentiation and development, the cellular response to extracellular effectors and stress, modulation of cell surface receptors and ion channels, DNA repair, regulation of the immune and inflammatory responses, and biogenesis of organelles (50). There are two successive steps of protein degradation by the ubiquitin system, including conjugation of multiple moieties of ubiquitin, and degradation of the tagged protein by the 26S proteasome. Ubiquitin can bind to the target protein by a series of ubiquitin promoter enzymes. Ubiquitin promoter includes a wide range of enzymes such as E1 ubiquitin activating enzymes, E2 ubiquitin binding enzymes, and E3 ubiquitin ligase enzymes. Ubiquitination is initiated by E1 which activates and transfers ubiquitin to E2. This E2 passes the ubiquitin to the corresponding E3 (50). In the present research, 0.5 mg/mL SBA treatment led to down-regulation of E1 only, while 2.0 mg/mL SBA treatment led to down-regulation of both the E1 and E2 to affect the ubiquitin mediated proteolysis process, and finally could regulate many processes such as cell cycle and apoptosis, etc.

### SBA Down-Regulated the AMPK Signal Transduction Mechanisms

AMPK, a central energy sensor, plays an important role on regulating cellular metabolism, and preserving cellular energy homeostasis. This sensor is involved in many cellular processes, including cell apoptosis (51). In the present research, down-regulated proteins (SIRT1, PP2A, FASN, SCD, ACC, and eEF2) and up-regulated protein (CPT1) were enriched in AMPK signal pathway in 2.0 mg/mL SBA treatment compared to control. SIRT1 is a key energy-sensing molecule in regulating mitochondrial biogenesis and can mutually regulate AMPK (52). The inhibition of SIRT1 induces growth arrest and apoptosis in several types of cancer cells (53). Park et al. (54) indicated that AMPK is negatively regulated by PP2A, which participates in regulating many important physiological processes, such as cell cycle, growth, apoptosis, and signal transduction, G1-S transition, DNA synthesis, and mitotic initiation (55). FASN plays an important role in regulating many cell processes. A related report indicated that the mitochondrial dysfunction is related to the inhibition of FASN, and consequently induces apoptosis (56). The activation of AMPK decreases protein synthesis through inhibition of eEF2, which plays a major role in protein synthesis and cell survival (57). Additionally, silencing of eEF2 expression increases mitochondrial elongation, cellular autophagy, and cisplatin sensitivity (58). Thus, the effect of SBA on cell cycle, apoptosis, and mitochondrial pathway was confirmed again.

### SBA Affected Subcellular Structure and Function

The results of GO annotation in proteomics indicated that SBA treated group had lower levels of proteins that involved in mitochondrial energy metabolism such as SDHB, and NDUFS6 etc. SDHB silencing increases reactive oxygen species (ROS) production (59). NDUFS6 plays an important role in regulating mitochondrial complex I activity and the mitochondrial apoptotic pathway in human malignant melanoma cells (60). These results also indicate a lower potential of the inner mitochondrial membrane (61). As described before, from the results of that SBA increased cell apoptotic rates, down-regulated the Bcl-2, up-regulated the Bax, and increased the level of cleaved caspases, including casp-9 and casp-3 activity. Such findings also suggested the destruction of the outer mitochondrial membrane. Therefore, the potential destruction of homeostasis of inner and outer mitochondrial membranes occurred in the SBA treatments.

Ribosome biogenesis is a complex regulated cellular process. Dysregulation of ribosome biogenesis or abnormal expression of ribosomal proteins (RPs) cause different disorders in the biological system (62). In addition, augmented ribosome biogenesis motivates a wide range of malignant tumors (63). In the present research, the differential expressed proteins in 2.0 mg/mL SBA treatment were also enriched in structural and functional ribosomal proteins, such as rpS12 (ribosomal protein S12); NAA10 (N-α-acetyltransferase 10); ribosomal protein L4 (rpL4); CYFIP2 (cytoplasmic fragile X mental retardation 1 interacting proteins 2), and FIP-2 [14 point seven (14.7K) Interaction Protein-2]. These proteins play significant roles in ribosome assembly, protein synthesis, cell cycle and cell apoptosis (64, 65). The RPS12 plays an essential role in cell growth and survival activities (66). The NAA10 is a common post-translational protein modification in eukaryotes, and the deficiency of NAA10 expression can induce cell cycle arrest and apoptosis (67, 68). The CYFIP2 is a p53-inducible gene, which inhibits many malignant processes such as colon cancer proliferation, caspase activation, and induce apoptosis (69). Therefore, with increasing SBA concentration, many structural and functional biological processes of mitochondria and ribosome, as well as protein synthesis and metabolism have been negatively affected.

## Conclusions

Soybean agglutinin blocked the cell cycle at G0/G1 phase and increased the cell apoptosis rates in IPEC-J2. The overall results of the cell survival activities and proteomic analysis indicated that the anti-nutritional mechanism of SBA is a complex cellular process, including DNA related processes such as replication, repairs and metabolism; protein translation and metabolism; signal-conducting relation; as well as subcellular structure and function. This study will provide more effective information for full understanding the mechanism of SBA toxicity.

## Data Availability Statement

The mass spectrometry proteomics data have been deposited to the ProteomeXchange Consortium via the PRIDE [1] partner repository with the dataset identifier PXD025751. http://www.ebi.ac.uk/pride/archive/projects/PXD025751.

## Author Contributions

LP and GQ designed the experiments. YL and LP performed the experiments. LP, HL, YZ, NB, and HS analyzed the data. LP and MF wrote the paper. All authors contributed to the article and approved the submitted version.

## Conflict of Interest

The authors declare that the research was conducted in the absence of any commercial or financial relationships that could be construed as a potential conflict of interest.
